# Bis(2-meth­oxy­benzyl­ammonium) di­aqua­bis­(di­hydrogen diphosphato-κ^2^ *O*,*O*′)cobaltate(II) dihydrate

**DOI:** 10.1107/S1600536814006102

**Published:** 2014-03-26

**Authors:** Adel Elboulali, Ahmed Selmi, Nicolas Ratel-Ramond, Mohamed Rzaigui, Samah Toumi Akriche

**Affiliations:** a Laboratoire de Chimie des Matériaux, Faculté des Sciences de Bizerte, 7021 Zarzouna Bizerte, Tunisia; b CEMES–CNRS, 29 rue Jeanne Marvig, 31055 Toulouse cedex 4, France

## Abstract

The title compound, (C_8_H_12_NO)_2_[Co(H_2_P_2_O_7_)_2_(H_2_O)_2_]·2H_2_O, crystallizes isotypically with its Mn^II^ analogue. It consists of alternating layers of organic cations and inorganic complex anions, extending parallel to (100). The complex cobaltate(II) anion exhibits -1 symmetry. Its Co^2+^ atom has an octa­hedral coordination sphere, defined by two water mol­ecules in apical positions and two H_2_P_2_O_7_^2−^ ligands in equatorial positions. The cohesion between inorganic and organic layers is accomplished by a set of O—H⋯O and N—H⋯O hydrogen bonds involving the organic cation, the inorganic anion and the remaining lattice water mol­ecules.

## Related literature   

For the isotypic Mn^II^ structure, see: Elboulali *et al.* (2013*b* ▶). For related structures with diphosphate units, see: Alaoui Tahiri *et al.* (2003 ▶); Essehli *et al.* (2005 ▶); Selmi *et al.* (2006 ▶, 2009 ▶); Ahmed *et al.* (2006 ▶); Gharbi *et al.* (1994 ▶); Gharbi & Jouini (2004 ▶); Elboulali *et al.* (2013*a* ▶). For distortion index calculations, see: Kobashi *et al.* (1997 ▶).
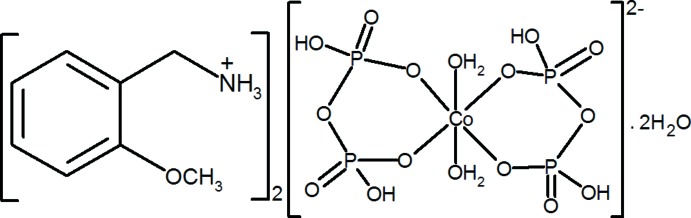


## Experimental   

### 

#### Crystal data  

(C_8_H_12_NO)_2_[Co(H_2_P_2_O_7_)_2_(H_2_O)_2_]·2H_2_O
*M* *_r_* = 759.28
Monoclinic, 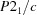

*a* = 14.050 (5) Å
*b* = 11.971 (5) Å
*c* = 9.161 (5) Å
β = 93.718 (5)°
*V* = 1537.6 (12) Å^3^
*Z* = 2
Mo *K*α radiation
μ = 0.85 mm^−1^
*T* = 293 K
0.25 × 0.19 × 0.13 mm

#### Data collection  

Nonius KappaCCD diffractometer
33422 measured reflections
4645 independent reflections
3135 reflections with *I* > 2σ(*I*)
*R*_int_ = 0.081

#### Refinement  

*R*[*F* ^2^ > 2σ(*F* ^2^)] = 0.096
*wR*(*F* ^2^) = 0.266
*S* = 1.08
4645 reflections
212 parameters
7 restraints
H atoms treated by a mixture of independent and constrained refinement
Δρ_max_ = 2.59 e Å^−3^
Δρ_min_ = −1.26 e Å^−3^

### 

Data collection: *COLLECT* (Hooft, 1998 ▶); cell refinement: *DIRAX/LSQ* (Duisenberg *et al.*, 2000 ▶); data reduction: *EVALCCD* (Duisenberg *et al.*, 2003 ▶); program(s) used to solve structure: *SHELXS97* (Sheldrick, 2008 ▶); program(s) used to refine structure: *SHELXL97* (Sheldrick, 2008 ▶); molecular graphics: *ORTEP-3 for Windows* (Farrugia, 2012 ▶) and *DIAMOND* (Brandenburg & Putz, 2005 ▶); software used to prepare material for publication: *WinGX* (Farrugia, 2012 ▶).

## Supplementary Material

Crystal structure: contains datablock(s) I. DOI: 10.1107/S1600536814006102/wm5011sup1.cif

Structure factors: contains datablock(s) I. DOI: 10.1107/S1600536814006102/wm5011Isup2.hkl

CCDC reference: 992564

Additional supporting information:  crystallographic information; 3D view; checkCIF report

## Figures and Tables

**Table 1:** Selected bond lengths (Å)

Co1—O1	2.075 (4)
Co1—O1*W*	2.095 (4)
Co1—O5	2.124 (4)

**Table 2:** Hydrogen-bond geometry (Å, °)

*D*—H⋯*A*	*D*—H	H⋯*A*	*D*⋯*A*	*D*—H⋯*A*
O3—H3⋯O2^i^	0.82	1.85	2.553 (6)	143
O6—H6⋯O7^ii^	0.82	1.77	2.571 (6)	166
O1*W*—H1*W*1⋯O2^iii^	0.86 (2)	1.98 (3)	2.827 (6)	168 (7)
O1*W*—H2*W*1⋯O7^ii^	0.86 (2)	2.00 (2)	2.851 (6)	171 (8)
N1—H1*A*⋯O2*W*	0.89	1.99	2.840 (8)	159
N1—H1*A*⋯O3^ii^	0.89	2.53	2.988 (7)	113
N1—H1*B*⋯O5^iv^	0.89	2.04	2.810 (7)	145
N1—H1*C*⋯O1	0.89	2.28	2.944 (7)	131
N1—H1*C*⋯O8	0.89	2.42	2.972 (9)	120
O2*W*—H1*W*2⋯O2^ii^	0.85 (2)	2.08 (4)	2.885 (7)	157 (9)
O2*W*—H2*W*2⋯O7^iii^	0.85 (2)	2.06 (4)	2.876 (7)	161 (9)

Symmetry codes: (i) 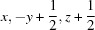; (ii) 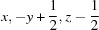; (iii) 
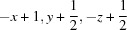
; (iv) 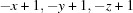.

**Fig. 1.**
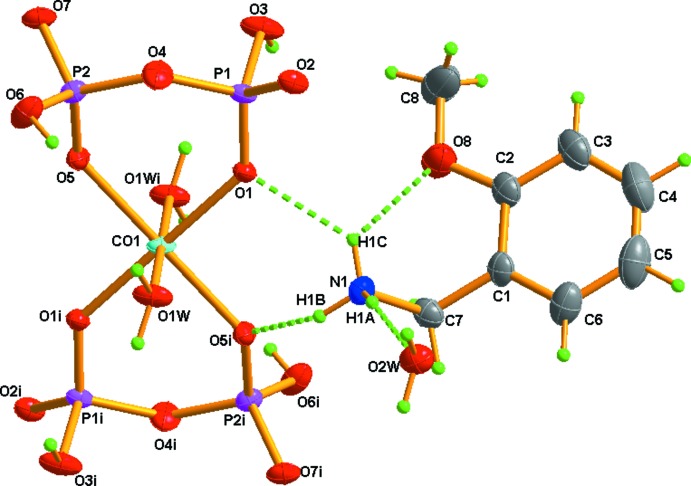
The molecular entities in the structure of (I) with the atom-labelling scheme. Displacement ellipsoids are drawn at the 30% probability level. H atoms are represented as small spheres of arbitrary radius. Hydrogen bonds are represented as dashed lines. [Symmetry code: (i) 1 - x, 1 - y, 1 - z.]

**Fig. 2.**
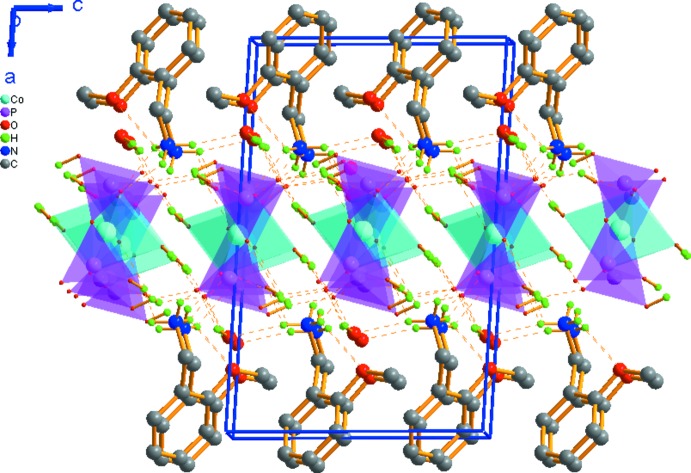
Perspective view of the crystal packing of (I) in a projection along [010]. The H-atoms not involved in hydrogen bonding were omitted.

## supplementary crystallographic information

### 1. Comment

As a part of our interest in diphosphate materials, we report here the preparation and the structural study of the title compound, (C_8_H_12_NO)_2_[Co(H_2_P_2_O_7_)_2_(H_2_O)_2_]·2H_2_O, (I), that crystallizes isotypically with its Mn^II^ analogue (Elboulali *et al.*, 2013*b*).

The asymmetric unit of (I) consists of a Co(II) atom, one H_2_P_2_O_7_^2-^ anion, one organic cation and two water molecules (one coordinating to Co(II) and the other a lattice water molecule). The Co(II) ion lies on an inversion centre, hence the complete formula unit is generated by this element of symmetry (Fig. 1).

The crystal structure of (I) exhibits the same type of architecture than that of the isotypic Mn^II^ analogue. It is built up from centrosymmetric [Co(H_2_P_2_O_7_)_2_(H_2_O)_2_]^2-^ complex anions arranged in layers parallel to (100). These laters are interconnected by a set of O—H···O and N—H···O hydrogen bonds (Table 2, Fig. 2) between the components.

The distortion index calculation (Kobashi *et al.*, 1997) of the CoO_6_ octahedron in the anion gives a value of 0.023, indicating a rather regular coordination sphere for this ion (radius 0.74 Å). The distortion index for the MnO_6_ octahedron in the isotypic Mn^II^ analogue is with 0.028 slightly greater, probably as a consequence of the larger ionic radius of Mn^II^ (0.80 Å). The Co—O bond lengths around Co^2+^ ion are between 2.075 (4) and 2.124 (4) Å (Table 1), similar to those observed in (NH_4_)_2_[Co(H_2_P_2_O_7_)_2_(H_2_O)_2_] (Essehli *et al.*, 2005) and due to the smaller ionic radius shorter than in [Mn(H_2_P_2_O_7_)_2_(H_2_O)_2_] units in related structures (Alaoui Tahiri *et al.*, 2003; Elboulali *et al.*, 2013*b*).

The P_2_O_7_ moiety has a quasi-eclipsed conformation with a mean O—P—O—P torsion angle of 19.6 ° and bridges the Co(II) ion through O1—P1 and O5—P2 linkages. The P_2_O_7_ group is bent, with a P1—O4—P2 bond angle of 132.9 (3)° as observed in other *M*(II)–organic diphosphate frameworks (Elboulali *et al.* 2013*a*; Selmi *et al.* 2006, 2009; Ahmed *et al.* 2006; Gharbi *et al.* 2004, 1994).

### 2. Experimental

Crystals of the title compound were obtained by the reaction of diphosphoric acid (2 mmol), CoCl_2_·6H_2_O (0.24 g; 1 mmol) and *2*-methoxybenzylamine (0.138 g; 1 mmol) carried out in an acidic medium. Diphosphoric acid, H_4_P_2_O_7_, was obtained from Na_4_P_2_O_7_ by using an ion-exchange resin (Amberlite IR 120).

### 3. Refinement

All H atoms attached to C, O and N atoms were fixed geometrically and treated as riding, with C—H = 0.93 Å with *U*_iso_(H) = 1.2*U*eq(C) for aromatic ring and C—H = 0.97 and 0.96 Å and N—H = 0.89 Å, respectively, for CH_2_, CH_3_ and NH_3_ units and O—H = 0.82 Å for the hydrogen diphosphate anion with *U*_iso_(H) = 1.5*U*_eq_(C, O or N). The water H atoms were refined using restraints [O—H = 0.85 (1) Å, H···H = 1.44 (2) A ° and *U*_iso_(H) = 1.5*U*_eq_(O)].

### Crystal data

**Table:**

(C_8_H_12_NO)_2_[Co(H_2_P_2_O_7_)_2_(H_2_O)_2_]·2H_2_O	*F*(000) = 786
*M**_r_* = 759.28	*D*_x_ = 1.640 Mg m^−^^3^
Monoclinic, *P*2_1_/*c*	Mo *K*α radiation, λ = 0.71073 Å
Hall symbol: -P 2ybc	Cell parameters from 33422 reflections
*a* = 14.050 (5) Å	θ = 2.2–30.9°
*b* = 11.971 (5) Å	µ = 0.85 mm^−^^1^
*c* = 9.161 (5) Å	*T* = 293 K
β = 93.718 (5)°	Prism, pink
*V* = 1537.6 (12) Å^3^	0.25 × 0.19 × 0.13 mm
*Z* = 2	

### Data collection

**Table:**

Nonius KappaCCD diffractometer	3135 reflections with *I* > 2σ(*I*)
Radiation source: fine-focus sealed tube	*R*_int_ = 0.081
Horizonally mounted graphite crystal monochromator	θ_max_ = 30.9°, θ_min_ = 2.2°
Detector resolution: 9 pixels mm^-1^	*h* = −20→18
ω and φ CCD rotation images, thick slices scans	*k* = −17→17
33422 measured reflections	*l* = −10→13
4645 independent reflections	

### Refinement

**Table:**

Refinement on *F*^2^	Primary atom site location: structure-invariant direct methods
Least-squares matrix: full	Secondary atom site location: difference Fourier map
*R*[*F*^2^ > 2σ(*F*^2^)] = 0.096	Hydrogen site location: inferred from neighbouring sites
*wR*(*F*^2^) = 0.266	H atoms treated by a mixture of independent and constrained refinement
*S* = 1.08	*w* = 1/[σ^2^(*F*_o_^2^) + (0.0687*P*)^2^ + 17.5972*P*] where *P* = (*F*_o_^2^ + 2*F*_c_^2^)/3
4645 reflections	(Δ/σ)_max_ = 0.003
212 parameters	Δρ_max_ = 2.59 e Å^−^^3^
7 restraints	Δρ_min_ = −1.26 e Å^−^^3^

### Special details

**Table:**

Geometry. All e.s.d.'s (except the e.s.d. in the dihedral angle between two l.s. planes) are estimated using the full covariance matrix. The cell e.s.d.'s are taken into account individually in the estimation of e.s.d.'s in distances, angles and torsion angles; correlations between e.s.d.'s in cell parameters are only used when they are defined by crystal symmetry. An approximate (isotropic) treatment of cell e.s.d.'s is used for estimating e.s.d.'s involving l.s. planes.
Refinement. Refinement of *F*^2^ against ALL reflections. The weighted *R*-factor *wR* and goodness of fit *S* are based on *F*^2^, conventional *R*-factors *R* are based on *F*, with *F* set to zero for negative *F*^2^. The threshold expression of *F*^2^ > σ(*F*^2^) is used only for calculating *R*-factors(gt) *etc*. and is not relevant to the choice of reflections for refinement. *R*-factors based on *F*^2^ are statistically about twice as large as those based on *F*, and *R*- factors based on ALL data will be even larger.

### Fractional atomic coordinates and isotropic or equivalent isotropic displacement parameters (Å^2^)

**Table:**

	*x*	*y*	*z*	*U*_iso_*/*U*_eq_	
Co1	0.5000	0.5000	0.5000	0.0248 (3)	
P1	0.58873 (11)	0.24678 (12)	0.46988 (14)	0.0240 (3)	
P2	0.38517 (11)	0.25622 (12)	0.51633 (14)	0.0249 (3)	
O1	0.5946 (3)	0.3708 (3)	0.4662 (4)	0.0278 (8)	
O2	0.6336 (3)	0.1843 (3)	0.3488 (4)	0.0302 (9)	
O3	0.6288 (4)	0.1972 (4)	0.6168 (5)	0.0471 (13)	
H3	0.6494	0.2477	0.6704	0.071*	
O4	0.4796 (4)	0.2083 (4)	0.4534 (6)	0.0446 (12)	
O5	0.3993 (3)	0.3764 (4)	0.5528 (5)	0.0309 (9)	
O6	0.3105 (4)	0.2386 (5)	0.3853 (5)	0.0434 (12)	
H6	0.3333	0.2586	0.3094	0.065*	
O7	0.3549 (4)	0.1825 (4)	0.6378 (4)	0.0349 (10)	
O1W	0.4616 (4)	0.4955 (4)	0.2753 (5)	0.0402 (11)	
H1W1	0.441 (6)	0.558 (3)	0.240 (8)	0.060*	
H2W1	0.435 (5)	0.438 (3)	0.233 (8)	0.060*	
O8	0.8402 (5)	0.3749 (6)	0.5079 (7)	0.0663 (18)	
N1	0.7205 (4)	0.4951 (5)	0.2793 (6)	0.0369 (12)	
H1A	0.7210	0.4784	0.1847	0.055*	
H1B	0.6716	0.5403	0.2934	0.055*	
H1C	0.7145	0.4327	0.3307	0.055*	
C1	0.8971 (5)	0.4798 (7)	0.3192 (8)	0.0454 (17)	
C2	0.9128 (6)	0.3901 (7)	0.4183 (9)	0.0514 (19)	
C3	0.9953 (7)	0.3286 (9)	0.4206 (12)	0.068 (3)	
H3A	1.0057	0.2708	0.4875	0.082*	
C4	1.0628 (8)	0.3536 (11)	0.3228 (15)	0.085 (4)	
H4	1.1191	0.3126	0.3253	0.101*	
C5	1.0490 (8)	0.4362 (13)	0.2235 (14)	0.087 (4)	
H5	1.0952	0.4509	0.1578	0.105*	
C6	0.9657 (7)	0.4997 (10)	0.2192 (11)	0.071 (3)	
H6A	0.9559	0.5555	0.1493	0.085*	
C7	0.8106 (5)	0.5514 (7)	0.3277 (9)	0.0474 (17)	
H7A	0.8066	0.5760	0.4280	0.057*	
H7B	0.8179	0.6173	0.2679	0.057*	
C8	0.8535 (9)	0.2951 (13)	0.6208 (15)	0.103 (5)	
H8A	0.8584	0.2220	0.5788	0.154*	
H8B	0.8003	0.2973	0.6812	0.154*	
H8C	0.9110	0.3118	0.6791	0.154*	
O2W	0.7511 (4)	0.4927 (5)	−0.0240 (6)	0.0487 (13)	
H1W2	0.732 (6)	0.435 (4)	−0.071 (9)	0.073*	
H2W2	0.732 (7)	0.555 (3)	−0.061 (9)	0.073*	

### Atomic displacement parameters (Å^2^)

**Table:**

	*U*^11^	*U*^22^	*U*^33^	*U*^12^	*U*^13^	*U*^23^
Co1	0.0412 (6)	0.0166 (5)	0.0165 (4)	0.0007 (4)	0.0011 (4)	0.0000 (4)
P1	0.0361 (8)	0.0222 (7)	0.0139 (5)	0.0062 (5)	0.0016 (5)	0.0004 (5)
P2	0.0336 (7)	0.0266 (7)	0.0144 (6)	−0.0044 (6)	0.0011 (5)	−0.0018 (5)
O1	0.031 (2)	0.0224 (19)	0.030 (2)	0.0035 (15)	0.0028 (16)	0.0026 (15)
O2	0.047 (3)	0.029 (2)	0.0150 (16)	0.0088 (17)	0.0041 (16)	−0.0026 (14)
O3	0.089 (4)	0.034 (3)	0.0170 (19)	0.015 (3)	−0.004 (2)	0.0004 (17)
O4	0.050 (3)	0.026 (2)	0.059 (3)	−0.002 (2)	0.009 (2)	−0.018 (2)
O5	0.032 (2)	0.028 (2)	0.034 (2)	0.0022 (16)	0.0104 (17)	−0.0002 (16)
O6	0.047 (3)	0.066 (3)	0.0172 (18)	−0.019 (2)	−0.0035 (18)	0.005 (2)
O7	0.057 (3)	0.029 (2)	0.0189 (18)	−0.0036 (19)	0.0000 (18)	0.0025 (15)
O1W	0.072 (3)	0.026 (2)	0.0205 (19)	0.002 (2)	−0.014 (2)	−0.0017 (16)
O8	0.058 (4)	0.087 (5)	0.052 (3)	−0.003 (3)	−0.001 (3)	0.022 (3)
N1	0.039 (3)	0.040 (3)	0.032 (3)	−0.001 (2)	0.006 (2)	−0.003 (2)
C1	0.035 (4)	0.059 (5)	0.041 (4)	−0.004 (3)	−0.006 (3)	−0.004 (3)
C2	0.039 (4)	0.058 (5)	0.054 (5)	−0.003 (3)	−0.012 (3)	−0.005 (4)
C3	0.051 (5)	0.073 (7)	0.078 (7)	0.003 (4)	−0.021 (5)	−0.013 (5)
C4	0.053 (6)	0.099 (9)	0.100 (9)	0.018 (6)	−0.006 (6)	−0.037 (8)
C5	0.062 (7)	0.130 (11)	0.072 (7)	−0.013 (7)	0.022 (5)	−0.036 (7)
C6	0.055 (6)	0.102 (8)	0.055 (5)	−0.016 (5)	0.007 (4)	−0.002 (5)
C7	0.047 (4)	0.046 (4)	0.048 (4)	0.002 (3)	−0.007 (3)	−0.002 (3)
C8	0.069 (7)	0.140 (12)	0.096 (9)	−0.025 (7)	−0.019 (6)	0.058 (9)
O2W	0.056 (3)	0.051 (3)	0.038 (3)	−0.005 (3)	−0.003 (2)	0.001 (2)

### Geometric parameters (Å, º)

**Table:**

Co1—O1^i^	2.075 (4)	N1—H1A	0.8900
Co1—O1	2.075 (4)	N1—H1B	0.8900
Co1—O1W^i^	2.095 (4)	N1—H1C	0.8900
Co1—O1W	2.095 (4)	C1—C6	1.393 (12)
Co1—O5	2.124 (4)	C1—C2	1.414 (12)
Co1—O5^i^	2.124 (4)	C1—C7	1.494 (11)
P1—O1	1.488 (4)	C2—C3	1.373 (12)
P1—O2	1.509 (4)	C3—C4	1.379 (17)
P1—O3	1.543 (4)	C3—H3A	0.9300
P1—O4	1.598 (5)	C4—C5	1.349 (18)
P2—O5	1.488 (5)	C4—H4	0.9300
P2—O7	1.503 (4)	C5—C6	1.393 (16)
P2—O6	1.556 (4)	C5—H5	0.9300
P2—O4	1.588 (5)	C6—H6A	0.9300
O3—H3	0.8200	C7—H7A	0.9700
O6—H6	0.8200	C7—H7B	0.9700
O1W—H1W1	0.86 (2)	C8—H8A	0.9600
O1W—H2W1	0.86 (2)	C8—H8B	0.9600
O8—C2	1.362 (11)	C8—H8C	0.9600
O8—C8	1.411 (12)	O2W—H1W2	0.85 (2)
N1—C7	1.477 (9)	O2W—H2W2	0.85 (2)
			
O1^i^—Co1—O1	180.0 (2)	C7—N1—H1B	109.5
O1^i^—Co1—O1W^i^	87.73 (18)	H1A—N1—H1B	109.5
O1—Co1—O1W^i^	92.27 (18)	C7—N1—H1C	109.5
O1^i^—Co1—O1W	92.27 (18)	H1A—N1—H1C	109.5
O1—Co1—O1W	87.73 (18)	H1B—N1—H1C	109.5
O1W^i^—Co1—O1W	180.000 (1)	C6—C1—C2	117.8 (8)
O1^i^—Co1—O5	92.42 (16)	C6—C1—C7	122.5 (8)
O1—Co1—O5	87.58 (16)	C2—C1—C7	119.7 (7)
O1W^i^—Co1—O5	85.82 (19)	O8—C2—C3	125.7 (9)
O1W—Co1—O5	94.18 (19)	O8—C2—C1	113.4 (7)
O1^i^—Co1—O5^i^	87.58 (16)	C3—C2—C1	120.9 (9)
O1—Co1—O5^i^	92.42 (16)	C2—C3—C4	119.3 (11)
O1W^i^—Co1—O5^i^	94.18 (19)	C2—C3—H3A	120.4
O1W—Co1—O5^i^	85.82 (19)	C4—C3—H3A	120.4
O5—Co1—O5^i^	180.00 (16)	C5—C4—C3	121.5 (11)
O1—P1—O2	116.8 (2)	C5—C4—H4	119.2
O1—P1—O3	112.7 (3)	C3—C4—H4	119.2
O2—P1—O3	107.7 (3)	C4—C5—C6	120.1 (11)
O1—P1—O4	109.8 (2)	C4—C5—H5	119.9
O2—P1—O4	103.4 (3)	C6—C5—H5	119.9
O3—P1—O4	105.4 (3)	C1—C6—C5	120.3 (11)
O5—P2—O7	116.2 (2)	C1—C6—H6A	119.9
O5—P2—O6	112.2 (3)	C5—C6—H6A	119.9
O7—P2—O6	106.4 (3)	N1—C7—C1	114.0 (6)
O5—P2—O4	109.2 (2)	N1—C7—H7A	108.7
O7—P2—O4	109.9 (3)	C1—C7—H7A	108.7
O6—P2—O4	102.0 (3)	N1—C7—H7B	108.7
P1—O1—Co1	134.7 (3)	C1—C7—H7B	108.7
P1—O3—H3	109.5	H7A—C7—H7B	107.6
P2—O4—P1	132.9 (3)	O8—C8—H8A	109.5
P2—O5—Co1	134.7 (2)	O8—C8—H8B	109.5
P2—O6—H6	109.5	H8A—C8—H8B	109.5
Co1—O1W—H1W1	114 (5)	O8—C8—H8C	109.5
Co1—O1W—H2W1	123 (5)	H8A—C8—H8C	109.5
H1W1—O1W—H2W1	114 (3)	H8B—C8—H8C	109.5
C2—O8—C8	117.6 (8)	H1W2—O2W—H2W2	116 (3)
C7—N1—H1A	109.5		
			
O2—P1—O1—Co1	136.9 (3)	O1W^i^—Co1—O5—P2	−116.7 (4)
O3—P1—O1—Co1	−97.5 (4)	O1W—Co1—O5—P2	63.3 (4)
O4—P1—O1—Co1	19.7 (4)	O5^i^—Co1—O5—P2	−67 (100)
O1^i^—Co1—O1—P1	−141 (100)	C8—O8—C2—C3	5.4 (14)
O1W^i^—Co1—O1—P1	88.9 (4)	C8—O8—C2—C1	−173.5 (9)
O1W—Co1—O1—P1	−91.1 (4)	C6—C1—C2—O8	−177.8 (8)
O5—Co1—O1—P1	3.2 (4)	C7—C1—C2—O8	4.2 (10)
O5^i^—Co1—O1—P1	−176.8 (4)	C6—C1—C2—C3	3.3 (12)
O5—P2—O4—P1	23.1 (6)	C7—C1—C2—C3	−174.7 (8)
O7—P2—O4—P1	−105.4 (5)	O8—C2—C3—C4	179.9 (9)
O6—P2—O4—P1	142.0 (5)	C1—C2—C3—C4	−1.3 (14)
O1—P1—O4—P2	−39.1 (6)	C2—C3—C4—C5	−0.8 (16)
O2—P1—O4—P2	−164.5 (5)	C3—C4—C5—C6	0.8 (18)
O3—P1—O4—P2	82.6 (5)	C2—C1—C6—C5	−3.3 (14)
O7—P2—O5—Co1	140.4 (4)	C7—C1—C6—C5	174.7 (9)
O6—P2—O5—Co1	−96.9 (4)	C4—C5—C6—C1	1.3 (17)
O4—P2—O5—Co1	15.5 (5)	C6—C1—C7—N1	111.2 (9)
O1^i^—Co1—O5—P2	155.7 (4)	C2—C1—C7—N1	−70.9 (9)
O1—Co1—O5—P2	−24.3 (4)		

Symmetry code: (i) −*x*+1, −*y*+1, −*z*+1.

### Hydrogen-bond geometry (Å, º)

**Table:**

*D*—H···*A*	*D*—H	H···*A*	*D*···*A*	*D*—H···*A*
O3—H3···O2^ii^	0.82	1.85	2.553 (6)	143
O6—H6···O7^iii^	0.82	1.77	2.571 (6)	166
O1*W*—H1*W*1···O2^iv^	0.86 (2)	1.98 (3)	2.827 (6)	168 (7)
O1*W*—H2*W*1···O7^iii^	0.86 (2)	2.00 (2)	2.851 (6)	171 (8)
N1—H1*A*···O2*W*	0.89	1.99	2.840 (8)	159
N1—H1*A*···O3^iii^	0.89	2.53	2.988 (7)	113
N1—H1*B*···O5^i^	0.89	2.04	2.810 (7)	145
N1—H1*C*···O1	0.89	2.28	2.944 (7)	131
N1—H1*C*···O8	0.89	2.42	2.972 (9)	120
O2*W*—H1*W*2···O2^iii^	0.85 (2)	2.08 (4)	2.885 (7)	157 (9)
O2*W*—H2*W*2···O7^iv^	0.85 (2)	2.06 (4)	2.876 (7)	161 (9)

Symmetry codes: (i) −*x*+1, −*y*+1, −*z*+1; (ii) *x*, −*y*+1/2, *z*+1/2; (iii) *x*, −*y*+1/2, *z*−1/2; (iv) −*x*+1, *y*+1/2, −*z*+1/2.
